# Development and validation of a nomogram for radiation-induced hepatic toxicity after intensity modulated radiotherapy for hepatocellular carcinoma: a retrospective study

**DOI:** 10.1093/jjco/hyae024

**Published:** 2024-02-19

**Authors:** Qiaoyuan Wu, Yudan Wang, Yuxin Wei, Zhengqiang Yang, Kai Chen, Jianxu Li, Liqing Li, Tingshi Su, Shixiong Liang

**Affiliations:** Department of Radiation Oncology, Guangxi Medical University Cancer Hospital, Nanning, China; Department of Radiation Oncology, Guangxi Medical University Cancer Hospital, Nanning, China; Department of Radiation Oncology, Guangxi Medical University Cancer Hospital, Nanning, China; Department of Interventional Therapy, National Cancer Center/National Clinical Research Center for Cancer/Cancer Hospital, Chinese Academy of Medical Sciences and Peking Union Medical College, Beijing, China; Department of Biostatistics and Data Science, The University of Texas Health Science Center at Houston, Houston, TX, USA, and Department of Biostatistics, The University of Texas MD Anderson Cancer Center, Houston, TX, USA; Department of Radiation Oncology, Guangxi Medical University Cancer Hospital, Nanning, China; Department of Radiation Oncology, Guangxi Medical University Cancer Hospital, Nanning, China; Department of Radiation Oncology, Guangxi Medical University Cancer Hospital, Nanning, China; Department of Radiation Oncology, Guangxi Medical University Cancer Hospital, Nanning, China

**Keywords:** radiation-induced hepatic toxicity, risk assessment model, hepatocellular carcinoma, dose–volume parameters, Child–Pugh score

## Abstract

**Objective:**

This study aimed to construct a nomogram to predict radiation-induced hepatic toxicity in patients with hepatocellular carcinoma treated with intensity-modulated radiotherapy.

**Methods:**

This study reviewed the clinical characteristics and dose–volume parameters of 196 patients with hepatocellular carcinoma. Radiation-induced hepatic toxicity was defined as progression of the Child–Pugh score caused by intensity-modulated radiotherapy. Factors relevant to radiation-induced hepatic toxicity were selected using receiver operating characteristic and univariate logistic analysis. A risk assessment model was developed, and its discrimination was validated.

**Results:**

Eighty-eight (44.90%) and 28 (14.29%) patients had radiation-induced hepatic toxicity ≥ 1 (Child–Pugh ≥ 1) and radiation-induced hepatic toxicity ≥ 2 (Child–Pugh ≥ 2). Pre-treatment Child–Pugh, body mass index and dose–volume parameters were correlated with radiation-induced hepatic toxicity ≥ 1 using univariate logistic analysis. V15 had the best predictive effectiveness among the dose–volume parameters in both the training (area under the curve: 0.763, 95% confidence interval: 0.683–0.842, *P* < 0.001) and validation cohorts (area under the curve: 0.759, 95% confidence interval: 0.635–0.883, *P* < 0.001). The area under the curve values of the model that was constructed by pre-treatment Child–Pugh, body mass index and V15 for radiation-induced hepatic toxicity ≥1 were 0.799 (95% confidence interval: 0.719–0.878, *P* < 0.001) and 0.775 (95% confidence interval: 0.657–0.894, *P* < 0.001) in the training and validation cohorts, respectively. Patients with a body mass index ≤ 20.425, Barcelona clinic liver cancer = C, Hepatitis B Virus-positive, Eastern Cooperative Oncology Group = 1–2 and hepatic fibrosis require lower V15 dose limits.

**Conclusions:**

Risk assessment model constructed from Pre-treatment Child–Pugh, V15 and body mass index can guide individualized patient selection of toxicity minimization strategies.

## Teaser

We constructed a risk assessment model for predicting radiation-induced hepatic toxicity by clinical factors and dosimetric variable, by which we found that V15 and body mass index were vital factors.

## Introduction

Hepatocellular carcinoma (HCC) is one of the most common malignancies worldwide, with the third-highest cancer-related mortality rate (1). According to the 2022 National Comprehensive Cancer Network guidelines and Chinese Society of Clinical Oncology, radiotherapy (RT) were recommended as the main treatment for patients with unresectable HCC and those who require adjuvant therapy (in cases of inadequate surgical margins, positive margins, postoperative pathology with microvascular invasion, lymph node metastasis, portal vein tumour thrombus, etc.) (2,3). Advanced radiotherapies, such as stereotactic body RT (SBRT), intensity-modulated RT (IMRT) and proton therapy, have become increasingly valuable for the clinical decision-making and treatment of unresectable HCC (4–8).

The risk of incidence of radiation-induced hepatic toxicity (RIHT) has historically limited the applicability of radiation for the treatment of liver malignancies (9,10). Especially, patients with larger tumours and poor baseline liver function may acquire the lethal radiation-induced liver disease (RILD) (11–13), which is classified into ‘classic’ and ‘non-classic’ RILD (14). RILD is the major form of dose-limiting toxicity associated with RT treatment in HCC (10). ‘Classic’ RILD includes anicteric hepatomegaly, ascites-elevated liver enzymes and alkaline phosphatase. ‘Non-classic’ RILD is more common and is characterized by significant elevation of serum transaminases (>5× upper limit of normal) and jaundice (15). The main type of RIHT is non-classic RILD because only a partial volume of the liver is currently irradiated at a high dose (16–18). There are many criteria for evaluating ‘non-classic’ RILD, such as Child–Pugh (CP) score, albumin-bilirubin score, and the common terminology criteria for adverse events (CTCAE) (18,19). Compared with CTCAE, the CP score correlates better with liver failure and overall survival (OS) and is more appropriate for patients with cirrhosis (15,16,20).

Many studies have found that RT, whether as adjuvant or radical treatment, improveed the overall survival rate of patients with liver cancer (21,22). However, this benefit will be offset once RILD occurs (23). A model to predict RIHT is urgently needed to avoid RIHT and thereby improve the survival of patients treated with RT for HCC.

In this study, we aimed to construct prediction models and nomograms of RIHT from IMRT treatment based on clinical factors and dose–volume parameters.

## Patients and methods

### Patients

This study retrospectively reviewed the clinical characteristics and dose–volume parameters of 481 patients with HCC at the Guangxi Medical University Cancer Hospital from February 2016 to August 2021. Finally, after eligibility and exclusion criteria, 196 patients with HCC were enrolled in this study (Fig. S1). All patients underwent IMRT. The eligibility criteria were as follows: (i) HCC treated with IMRT to the liver site; (ii) CP-A or -B class; (iii) Eastern Cooperative Oncology Group (ECOG) scores of 0–2; (iv) no contraindication to RT and estimated survival greater than 3 months after RT; (v) no intolerance or interruption of RT for more than a week; (vi) patients who stipulate a minimum of 1 month post the completion of other treatments and must have recovered and stabilized liver function after the other treatments before undergoing radiotherapy; and (vii) complete follow-up information. The exclusion criteria were as follows: (i) liver metastases and other pathological types of liver tumours; (ii) CP-C class; (iii) ECOG score > 2; (iv) incomplete follow-up information; and (v) RT interruption.

### Radiation treatment

Patients were placed in a supine position with their arms overhead during free quiet breathing, fixed in a negative pressure vacuum body mould, and contrast-enhanced computed tomography was performed. For patient ineligible for surgery, the gross tumour volume (GTV) delineation included the boundaries of the tumours, metastatic lymph nodes and venous tumour thrombus. For postoperative patients with positive margins, the residual area corresponded to the GTV region. The clinical target volume (CTV) was defined as GTV plus a 4–5 mm margin. In patients receiving postoperative adjuvant radiotherapy, there was no GTV, and the CTV was defined by expanding 5–10 mm beyond the postoperative margins. The planning target volume included the CTV plus a 5–10 mm margin, implemented to account for respiratory motion and setup uncertainty (8,24,25). All target areas and organs at risk were contoured and calculated using the Pinnacle 3 system (Philips, Netherlands) or the MIM 6.8 system (MIM, USA). IMRT plans were designed using the Monaco treatment planning system (version 5.1) or the Pinnacle 3 system (Philips, Netherlands). A total irradiation dose of 45–60 Gy in 15–30 fractions was administered using a linear accelerator with 6 MV X-rays (ELEKTA Versa-HD or ELEKTA Synergy, Sweden).

### Follow-up and evaluation of hepatic toxicity

All patients were followed up at the first, third and sixth month after the completion of RT. Physical examination and blood tests before RT [such as aspartate aminotransferase (AST), alanine aminotransferase (ALT), bilirubin, serum albumin, prothrombin time, alkaline phosphatase and alpha-fetoprotein levels] were performed at every visit to assess hepatic toxicity. The serum ALT and AST levels were determined using commercial kits by an automatic blood biochemical analyser (3500 rpm for 15 min to separate the serum at 4°C).

### Statistical analysis and prediction model

We calculated the sample size through PASS software. Power ≥ 0.8, α ≤ 0.05, the minimum required sample size was determined to be 194. Divide the entire cohort into training or validation cohorts by random sampling method (ratio, 3:1). The primary endpoint, RIHT ≥1(CP ≥ 1) or ≥ 2 (CP ≥ 2), was defined as a progression in CP score of at least 1 or 2 points, respectively, within 6 months of RT completion. Fisher’s exact test, *χ*^2^ test and Wilcoxon test were used to analyse the training and validation cohorts. Correlations between the clinical and dose–volume parameters were assessed using Spearman’s rank test (correlation coefficient *r* > 0.2 indicates a correlation). Factors associated with the risk of RIHT were selected using univariate analysis in the training cohort (*P* < 0.05 indicates statistical significance). Factors that were statistically significant and not correlated with each other were subjected to a multivariate analysis to construct the model.

Risk assessment models and nomograms were developed and validated using the area under the curve (AUC). The cut-off point for each optimal predictor was obtained using X-tile.

All statistical analyses were performed using SPSS (version 25.0; SPSS Institute, Chicago, IL, USA) and R version 4.0.5 (http://www.r-project.org/), and statistical significance was set at *P* < 0.05.

## Results

### Patient characteristics

**Figure 1:**
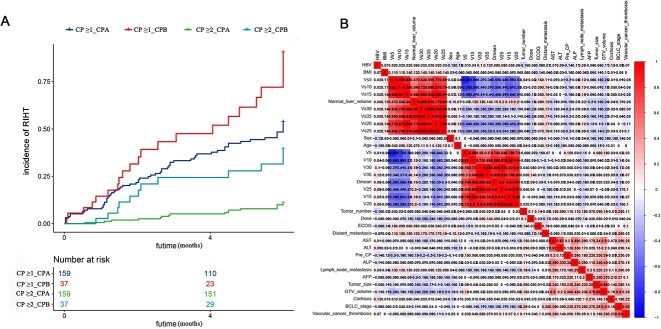
Incidence of radiation-induced hepatic toxicity (RIHT) and factors correlation heat map by the Spearman’s rank test. (A) The incidence of RIHT with time in patients with different Child–Pugh (CP) classes. (B) Correlation analysis between all clinical factors and dose–volume parameters. The correlation coefficients between the dose–volume parameters were all greater than 0.2. Vx, the percentage [%] of normal liver volume receiving more than x Gy. CP ≥ 1, the increase in Child–Pugh score of 1 point or more; CP ≥ 2, the increase in Child–Pugh score of 2 points or more; RIHT, radiation-induced hepatic toxicity; CP, Child–Pugh.

A flowchart of this process is presented in Fig. S1. A total of 196 patients were enrolled (training cohort, 137 patients; validation cohort, 59 patients). Eighty-eight (44.90%) and twenty-eight (14.29%) patients experienced RIHT ≥1 and RIHT ≥2, respectively. Fifty-eight (65.90%) patients developed RIHT after CP evaluation within 3 months (Fig. 1A). Patients with pre-treatment CP (Pre-CP) -B class were more likely to develop RIHT (Fig. 1A). RIHT = 1 occurred in 50 (31.4%) and RIHT ≥2 occurred in 16 (10.06%) of the Pre-CP A class patients. RIHT = 1 occurred in 10 (27.03%) and RIHT ≥2 occurred in 12 (32.43%) of the Pre-CP B class patients (Fig. 1A).

There were no significant differences between the training and validation cohorts in factors such as body mass index (BMI), Pre-CP score, dose–volume parameters and the number of people infected with Hepatitis B Virus (HBV) DNA. The patient characteristics were shown in Tables 1 and 2.

**Table 1:** Baseline tables for the training cohort and validation cohort

	Training cohort	Validation cohort	P
Sex			0.705
Male	123 (89.8%)	54 (91.5%)	
Female	14 (10.2%)	5 (8.5%)	
Age, years			0.092
≤55	76 (55.5%)	25 (42.4%)	
>55	61 (44.5%)	34 (57.6%)	
HBV			0.25
Negative	32 (23.4%)	19 (32.2%)	
Positive	105 (76.6%)	40 (67.8%)	
Cirrhosis			0.329
Yes	80 (58.4%)	30 (50.8%)	
No	57 (41.6%)	29 (49.2%)	
ECOG			0.877
0	22 (16.1%)	10 (16.9%)	
1/2	115 (83.9%)	49 (83.1%)	
Aspartate aminotransferase, u/l			0.334
≤40	73 (53.3%)	27 (45.8%)	
>40	64 (46.7%)	32 (54.2%)	
Alanine aminotransferase, u/l			0.079
≤40	72 (52.6%)	39 (66.1%)	
>40	65 (47.4%)	20 (33.9%)	
Aspartate aminotransferase, u/l			0.707
≤150	101 (73.7%)	45 (76.3%)	
>150	36 (26.3%)	14 (23.7%)	
AFP, ng/ml			0.375
<400	97 (70.8%)	38 (64.4%)	
≥400	40 (29.2%)	21 (35.6%)	
BCLC			0.931
A	32 (23.4%)	14 (23.7%)	
B	14 (20.2%)	5 (8.5%)	
C	91 (66.4%)	40 (67.8%)	
Tumour size, cm			0.543
≤5	54 (39.4%)	26 (44.1%)	
>5	83 (60.6%)	33 (55.9%)	
Lymph_node_metastasis			0.421
No	112 (81.8%)	51 (86.4%)	
Yes	25 (18.2%)	8 (13.6%)	
Distant_metastasis			0.255
No	116 (84.7%)	46 (78.0%)	
Yes	21 (15.3%)	13 (22.0%)	
Vascular_cancer_thrombosis			0.548
No	82 (59.9%)	38 (64.4%)	
Yes	55 (40.1%)	21 (35.6%)	
Immune checkpoint inhibitor therapy			0.545
No	122 (89.1%)	49 (86.0%)	
Yes	15 (10.9%)	8 (14.0%)	
Targeted therapy			0.228
No	131 (95.6%)	52 (91.2%)	
Yes	6 (4.4%)	5 (8.8%)	
Pre-CP			0.459
≤6	113 (82.5%)	46 (78.0%)	
>6	24 (17.5%)	13 (22.0%)	
CP ≥ 1			
Yes	56 (40.9%)	32 (54.2%)	0.085
No	81 (59.1%)	27 (45.8%)	
CP ≥ 2			0.253
Yes	17 (12.4%)	11 (18.6%)	
No	120 (87.6%)	48 (81.4%)	

Both cohorts were well balanced in the clinical characteristics. Statistical comparisons between the training and validation cohorts were computed using the *χ*^2^ test. HBV, Hepatitis B Virus; ECOG, Eastern Cooperative Oncology Group; AFP ; BCLC, Barcelona clinic liver cancer; CP, Child–Pugh.

**Table 2:** Dose–volume parameters baseline tables for the training cohort and validation cohort

	Training cohort median (IQR)	Validation cohort median (IQR)	Z	P
Dose, cGy	51 (50,60)	56 (50,60)	−0.659	0.51
GTV_volume, ml	192.92 (55.95642.24)	183.91 (83.11609.30)	−0.136	0.892
Normal_liver_volume, ml	971.95 (805.871112.19)	1082.20 (715.511282.24)	−0.824	0.41
Dmean, cGy	1757.00 (1355.602120.80)	1728.80 (1199.602027.00)	−1.47	0.142
V5, %	67.12 (57.86,83.56)	68.73 (55.63,74.00)	−1.142	0.253
V10, %	51.07 (36.74,62.79)	47.71 (36.82,56.97)	−1.281	0.2
V15, %	41.00 (28.98,51.08)	35.75 (25.58,49.12)	−1.374	0.169
V20, %	33.84 (24.08,43.46)	32.16 (20.49,41.90)	−1.484	0.138
V25, %	28.61 (20.31,37.61)	27.70 (17.93,33.80)	−1.353	0.176
V30, %	24.19 (15.94,31.15)	23.23 (14.78,29.87)	−0.921	0.357
V35, %	19.68 (12.05,25.48)	18.75 (11.01,25.41)	−0.641	0.522
Vs5, ml	296.57 (155.50405.82)	308.38 (235.76468.05)	−1.524	0.128
Vs10, ml	464.87 (317.88612.87)	474.69 (365.33,660.78)	−1.456	0.145
Vs15, ml	550.23 (434.28685.33)	595.25 (476.00786.46)	−1.703	0.088
Vs20, ml	613.68 (504.34760.71)	656.00 (525.41839.67)	−1.541	0.123
Vs25, ml	676.81 (559.66824.27)	760.95 (562.20899.02)	−1.366	0.172
Vs30, ml	720.51 (614.66858.76)	799.86 (582.98957.82)	−1.064	0.287
Vs35, ml	770.60 (656.61917.87)	822.00 (599.371041.98)	−1.061	0.289
BMI, kg/m^2^	22.20 (19.90,24.75)	21.50 (19.95,23.40)	−0.743	0.458

Both cohorts were well balanced in the dose–volume parameters variables. Statistical comparisons between the training and validation cohorts were computed using the Wilcoxon test. IQR, interquartile range; BMI, body mass index; Vx, the percentage [%] of normal liver volume receiving more than x Gy; Vsx, the volume of normalliver receiving more than xGy.

To avoid model overfitting, the factors were filtered using Spearman’s rank test. This test was used to assess the correlations between any two factors. If the correlation coefficient is >0.2, the two variables are correlated and cannot be used to construct a model simultaneously. We found a strong correlation between the dose–volume parameters (Fig. 1B).

### Univariate logistic analysis of all factors and predictive effectiveness of dose–volume parameters

**Figure 2:**
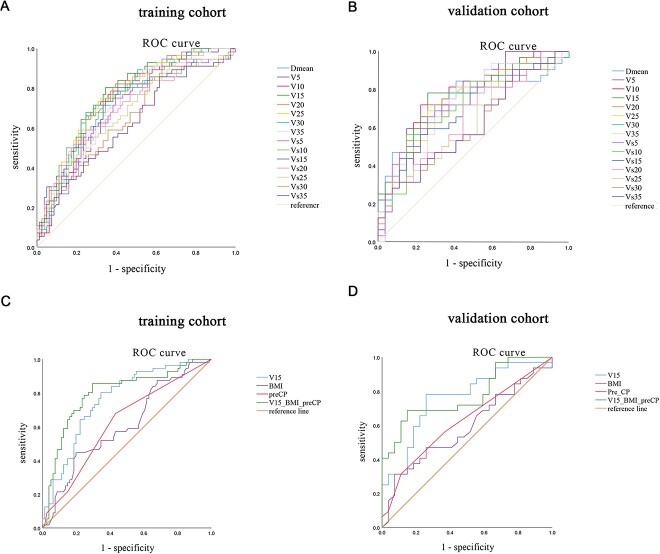
Univariate analysis of risk with RIHT and construction of the multivariate logistic regression model. (A) Receiving operator characteristic (ROC) curves for different dosimetric parameters in training cohort (V15 has the best prediction performance). (B) ROC curves for different dosimetric parameters in validation cohort (V15 has the best prediction performance). (C) ROC curves for single variables and the model in training cohort (this model was constructed by V15, body mass index (BMI), and pre-CP through logistic multivariate regression). (D) ROC curves for single variables and the model in validation cohort (this model was constructed by V15, BMI, and pre-CP through logistic multivariate regression). RIHT, radiation-induced hepatic toxicity. Vx, the percentage [%] of normal liver volume receiving more than x Gy; Vsx, the volume of normalliver receiving more than xGy. V15-BMI-preCP, this model was constructed by V15, BMI and pre-CP through logistic multivariate regression.

Subsequently, univariate logistic analysis was performed for all variables to predict factors related to RIHT ≥ 1. The results showed that dose–volume parameters, BMI, and Pre-CP scores were significantly correlated with RIHT ≥ 1 (Table S1). Receiving operator characteristic (ROC) curves were used to assess the predictive effectiveness of each dose–volume parameter for predicting RIHT ≥ 1. The results showed that the sensitivity and specificity of dose–volume parameters for predicting RIHT were high, with V15 (the percentage of normal liver volume receiving > 15 Gy) being the highest. The AUC value for V15 was 0.763 (95% confidence interval [CI]: 0.683–0.842, *P* < 0.001) in the training cohort (Fig. 2A), and 0.759 (Fig. 2B) in the validation cohort (95% CI: 0.635–0.883, *P* < 0.001) (Table S2).

### Prediction model and nomogram

In the training cohort, we used factors that were statistically significant in univariate logistic analysis (BMI, Pre-CP and V15) to construct a multivariate logistic regression analysis model for RIHT (Table S1). We found that in the training cohort, the AUC values for BMI, Pre-CP and model that was constructed by BMI, Pre-CP and V15 were 0.609 (95% CI: 0.513–0.705), 0.623 (95% CI: 0.528–0.718) and 0.799 (95% CI: 0.719–0.878), respectively (Fig. 2C). In the validation cohort, the AUC values for BMI, Pre-CP and model (BMI, Pre-CP and V15) were 0.586 (95% CI: 0.440–0.723), 0.623 (95% CI: 0.481–0.766) and 0.775 (95% CI: 0.657–0.894), respectively (Fig. 2D).

**Figure 3:**
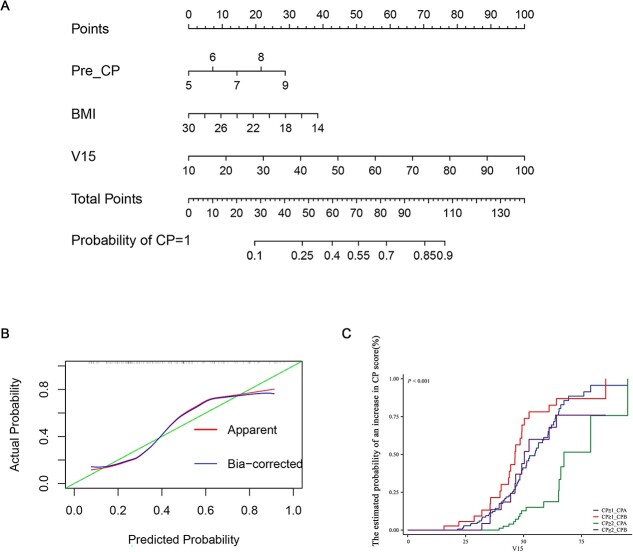
Construction of nomograms and validation of model efficacy. (A) Nomograms of the model. (B) Calibration curves of the model. (C) The probability of RIHT increased in a sigmoid manner with an increase in V15 (%) in patients with CP-A and -B class. V15, the percentage [%] of normal liver volume receiving more than 15 Gy; RIHT, radiation-induced hepatic toxicity; CP ≥ 1, the increase in Child–Pugh score of 1 point or more; CP ≥ 2, the increase in Child–Pugh score of 2 points or more.

A nomogram was constructed from V15, Pre-CP and BMI to visually predict more accurately the probability of RIHT (Fig. 3A). The calibration curve showed that the RIHT predicted by the model was consistent with the actual RIHT (Fig. 3B).

### Incidence of RIHT in different subgroups

The estimated probability values of the RIHT for CP ≥ 1 or CP ≥ 2 according to V15 in various Pre-CP classes were shown in Fig. 3C. The results found that more careful V15 limitation should be required in patients with CP-B class. The probability of RIHT increased in a sigmoid manner with an increase in V15 (%) in patients with CP-A and -B class.

### Limiting doses of V15 for different clinical factors

In order to individualize the treatment of patients, we further explored the dose limitation of V15 in patients with different clinical factors using X-tile. The results revealed that patients with BMI ≤ 20.425 (the dose limitation of V15 < 34.90%), ECOG = 1–2 (<33.67%), Alpha-fetoprotein (AFP) ≥ 400 ng/ml (<33.20%), Barcelona clinic liver cancer (BCLC) = C (<39.45%),  HBV -positive (<34.90%) and hepatic fibrosis (<34.90%) required a lower dose of V15 (Table S3).

## Discussion

RIHT is a serious, dose-limiting toxicity in patients with HCC receiving RT. Although most cases are usually self-limiting and resolve with supportive therapy, serious complications may lead to deterioration of the liver reserve, and in severe cases, liver failure and death (26). Different definitions of RIHT have been used for liver SBRT. There have been even fewer studies on IMRT. Therefore, this study included patients treated with IMRT to assess the criteria and parameters that were clinically relevant for defining RIHT in patients with HCC. The establishment of this prediction model may enable different patients to enjoy individualized treatment and avoid patients who are not prone to RIHT being limited in radiotherapy dose due to uniform criteria, which results in insufficient radiotherapy dose to the tumour target area.

The definition of RIHT ≥1 as progression in CP score of more than 1 point was not used in any previous studies in the IMRT era. Almost all studies have described CP ≥ 2 as non-classic RILD (18,19,26,27). Therefore, to improve the survival of patients after RT, RIHT ≥1 should be an important observational endpoint. This conclusion was consistent with the results of a prospective study conducted during SBRT (20).

Due to the invasiveness and high risk of liver biopsies after RT, most studies have evaluated RIHT based on clinical symptoms and haematological test results. However, substantial evidence suggested that clinicians tend to underestimate or miss symptomatic adverse events. In the study of Tae et al., of the 105 patients, Grade 1 RIHT was observed in 21 patients (20.0%), Grade 2 in 7 patients (6.7%), Grade 3 in 5 patients (4.8%) and Grade 4 in 1 patient (1.0%) (26). Similarly, in a study by Stenmark et al., among 48 patients who received partial liver irradiation at a median dose of 55 Gy, three patients (6%) developed RILD (28). To further investigate the effect of different RT techniques on the incidence of classic RILD, Sun et al. enrolled 40 patients with HCC treated with IMRT, proton RT (PRT) or carbon-ion RT (CIRT). The results of the classic RILD incidences (%) were 22.3 ± 30.0 in IMRT, 2.3 ± 4.9 in PRT and 1.2 ± 2.4 in CIRT (29). In the retrospective study of our team, 109 patients completed three-dimensional conformational RT (3D-CRT) treatment, 16% of whom developed RILD (30). Subsequently, our team then followed up patients with class A CP where 7.9% developed RILD (31). In these studies, the RIHT incidence was relatively low. The most likely reason for this was that RIHT ≥1 within 6 months was used as the first endpoint in our study, unlike other studies in which RIHT ≥2 (in our study, 14.29%) was widely used as the endpoint (19). Second, we enrolled patients with all grades of liver function, and there were more patients with cirrhosis in this study, which could have increased the incidence of RIHT (32). Therefore, we believe that RIHT ≥1 should also be used as an observed endpoint for RIHT.

In patients with unresectable HCC or those who require adjuvant RT after surgery, it is necessary to understand the predictors of RIHT using IMRT. In our previous study, we constructed a prognostic model of RIHT using dose–volume parameters. The AUC of V15 was 0.803 (95% CI: 0.703–0.881) with a cut-off value of 33.1% (sensitivity, 65.00%; specificity, 86.15%), and the AUC of Vs10 was 0.832 (95% CI: 0.736–0.905) with a cut-off value of 416.2 ml (sensitivity, 65.00%; specificity, 90.77%) (20). In this study, the AUC values (0.799 [95% CI: 0.719–0.878, *P* < 0.001] and 0.775 [95% CI: 0.657–0.894, *P* < 0.001], and the cut-off values of V15 (39.458%) and Vs10 (434.61 ml) were consistent with our previous findings, although our prospective study aimed to explore hepatic injury in SBRT. The model predicted an AUC value of 0.806 (95% CI: 0.729–0.883, *P* < 0.001) for RIHT ≥2. The tumour size of IMRT-treated patients varies greatly among populations, and their target areas consistency were poorer than that of SBRT, which reduceed the predictive efficacy of our models. Some scholars use more indicators as criteria for the RIHT, and its more complicated inclusion criteria lead to a potentially significant bias and variability (33). Few studies have predicted RIHT caused by conformal RT. A Korean study reported a statistically significant difference in the probability of RIHT between patients with HCC treated with 3D-CRT and those treated with IMRT (*P* = 0.007) (34). However, in an era where IMRT is dominant, the probability of RIHT remains unclear.

RIHT is a dose-limiting toxicity. Most studies have found V30 and mean liver dose to be important dosimetric parameters (18,26,30,35). However, the populations in these studies were limited by disease stage and grade. We constructed models using dosimetric parameters and clinical factors and found that V15 had the best predictive performance, which was consistent with our previous research (20). The CP score is an important criterion for predicting prognosis. Our previous results showed that among 109 patients with HCC treated with 3D-CRT, RILD occurred in 9 of 16 patients with class B CP (56%) compared with 8 of 93 patients with class A CP (9%), and the severity of liver dysfunction was the only independent predictor of RILD in a multivariate analysis (30). Li et al. treated 62 patients with gastric cancer using 3D-CRT and found that V35 and ALP levels were significant predictive factors for RIHT (36). The reason why the Pre-CP level was not used as a predictive variable in the study by Li et al. is that HCC is usually associated with poor liver function and cirrhosis. In addition, patients with poor liver function (Class B) appear to be less tolerant of radiation (33,37,38). The study was consistent with our findings that Pre-CP class B was more likely to trigger RIHT ≥2. The BMI is a nutritional indicator that is closely related to the adverse effects of radiotherapy. Koide et al. found that among the parameters, vital capacity and BMI showed the strongest negative correlation with the mean heart dose (*r* = −0.33) and mean lung dose (*r* = −0.34), respectively (39,40). Therefore, a high BMI can reduce the incidence of RIHT.

This study has several limitations. First, the progression in the CP score as a criterion for RIHT can lead to certain subjectivity, such as ascites and hepatic encephalopathy. Second, our eligibility criteria included palliative RT owing to the large size of the tumour, which can lead to an increased incidence of RIHT. We lacked external validation to further validate the models. In addition, we predicted RIHT based only on clinical factors and dose–volume parameters. The accuracy of the model will be further improved if multi-omics, such as radiomics, are combined to construct a model to predict RIHT. In the future, we will construct a radiomics-combined prediction model and collect other information from other centers or implement prospective studies to validate the accuracy of the model. Finally, the retrospective nature of this study represented a major limitation. Additionally, we are planning to undertake a prospective study in the future to validate the stability of our model.

## Conclusions

In conclusion, we were able to accurately predict RIHT based on clinical factors and dose–volume parameters with high sensitivity and specificity. We suggested that patients with BMI ≤ 20.425, ECOG = 1–2, AFP ≥ 400 ng/ml, BCLC = C, HBV-positive and hepatic fibrosis required a lower dose of V15.

## Supplementary Material

Table_S1_hyae024

Table_S2_hyae024

Table_S3_hyae024

Fig_S1_hyae024

## Data Availability

The datasets used and/or analysed during the current study are available from the corresponding author on reasonable request.

## Authors’ responsibility

This manuscript has not been published or presented elsewhere in part or in entirety and is not under consideration by another journal. The study design was approved by the appropriate ethics review board and the requirement of informed consent was waived. We have read and understood your journal’s policies, and we believe that neither the manuscript nor the study violates any of these. We have obtained all necessary permissions to publish any figures or tables in the manuscript, and we have assured that the authors will pay for any necessary charges. All authors have approved the manuscript for submission.
