# Supplementary figures and images for: Development and validation of a nomogram for radiation-induced hepatic toxicity after intensity modulated radiotherapy for hepatocellular carcinoma: a retrospective study
Source: Jpn J Clin Oncol. 2024 Feb 19;54(6):699–707. doi: 10.1093/jjco/hyae024 (PMC11144290; doi:10.1093/jjco/hyae024)

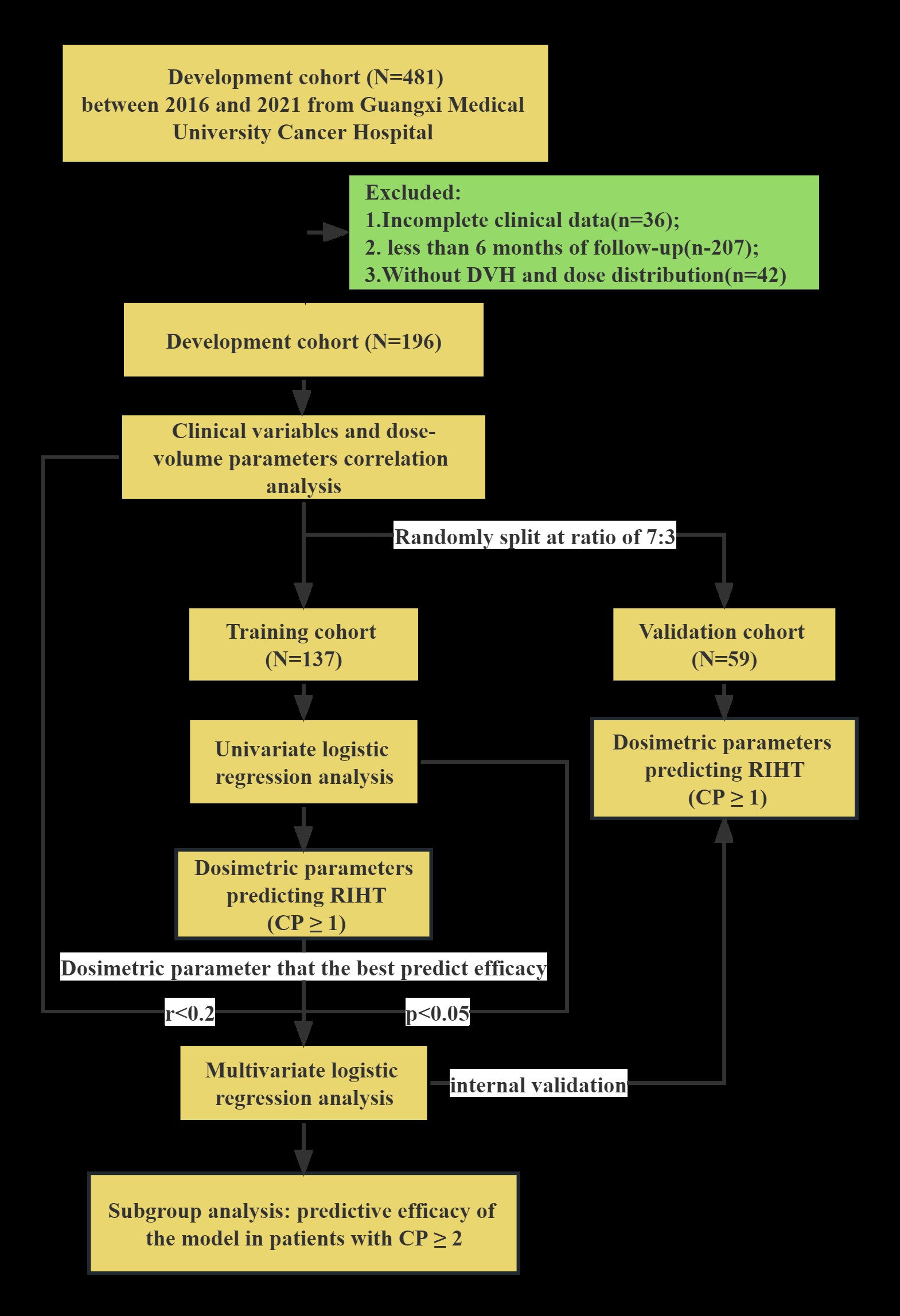

Supplement: Fig_S1_hyae024 [file fig_s1_hyae024.jpeg]
